# The relationship between the ascending aortic and left ventricular size after aortic valve replacement

**DOI:** 10.1186/1749-8090-10-S1-A302

**Published:** 2015-12-16

**Authors:** Ingrid Schusterova, Panagiotis Artemiou, Martina Polackova, Frantisek Sabol, Alzbeta Tohatyova

**Affiliations:** 1 East Slovakian Cardiovascular Institute, Kosice, Slovakia; 2 Department of Paediatrics and Adolescent Medicine, Faculty of Medicine, Pavol Jozef Safarik University in Kosice, Slovakia

## Background/Introduction

Different studies in the literature examined the fate of the ascending aorta and aortic root after valve replacement and various factors such as bicuspid aortic valve and aortic valve pathology were studied. The effect of the left ventricular end diastolic diameter (LVDD) has not been yet examined.

## Aims/Objectives

In this retrospective study we analyze for the first time the effect of the left ventricular end-diastolic diameter and its role on the aortic expansion after aortic valve replacement one year after the procedure.

## Methods

Forty three consecutive patients took part in this study. All of them underwent aortic valve replacement (AVR) in our Institution between the years 2012-2013 with either mechanical or biological prosthetic aortic valve.

## Results

After aortic valve replacement there was a decrease in the ascending aorta and aortic root dimensions (p = 0.001, p = 0.001 respectively). Left ventricular end-diastolic diameter and ejection fraction did not changed significantly after AVR. There were the correlations between the preoperative ascending aortic size and the preoperative and after the first year left ventricular end-diastolic diameter (r = 0.419, p = 0.001 and r = 0.320, p = 0.314 respectively). Postoperatively there was also a correlation between the ascending aortic size and the preoperative and after the first year left ventricular end-diastolic diameter (r = 0.320, p = 0.003 and r = 0.136, p = 0.335 respectively).

## Discussion/Conclusion

In conclusion, in this retrospective study we showed that there was a correlation between the preoperative and postoperative aortic size (ascending aorta and aortic root) and the postoperative left ventricular end-diastolic diameter. Although it is early for definite clinical conclusions, however it is the first study which tried to analyze this relationship.

